# Comparison of the Burden and Temporal Pattern of Hospitalisations Associated With Respiratory Syncytial Virus (RSV) Before and After COVID‐19 in New Zealand

**DOI:** 10.1111/irv.13346

**Published:** 2024-07-09

**Authors:** Nikki Turner, Nayyereh Aminisani, Sue Huang, Jane O'Donnell, Adrian Trenholme, David Broderick, Janine Paynter, Lorraine Castelino, Cameron Grant, Peter McIntyre

**Affiliations:** ^1^ Department of General Practice and Primary Healthcare University of Auckland Auckland New Zealand; ^2^ Institute of Environmental Science and Research ESR Wellington New Zealand; ^3^ Department of Anaesthesiology University of Auckland Auckland New Zealand; ^4^ Kidz First Childrens Hopsital Te Whatu Ora – Health New Zealand Counties Manukau Auckland New Zealand; ^5^ Department of Paediatrics: Child & Youth Health University of Auckland Auckland New Zealand; ^6^ Starship Children's Hospital Te Whatu Ora – Health New Zealand Te Toka Tumai Auckland Auckland New Zealand; ^7^ Department of Women's and Children's Health University of Otago Dunedin New Zealand

**Keywords:** epidemic, outbreak, respiratory syncytial virus (RSV), SARI

## Abstract

**Background:**

Changes in the epidemiology of illnesses caused by respiratory syncytial virus (RSV) infection following the COVID‐19 pandemic are reported. The New Zealand (NZ) COVID‐19 situation was unique; RSV community transmission was eliminated with the 2020 border closure, with a rapid and large increase in hospitalizations following the relaxation of social isolation measures and the opening of an exclusive border with Australia.

**Methods:**

This active population‐based surveillance compared the age‐specific incidence and seasonality of RSV‐associated hospitalizations in Auckland, NZ, for 2 years before and after the 2020 border closures. Hospitalisation rates between years were compared by age, ethnicity (European/other, Māori, Pacific and Asian) and socioeconomic group (1 = *least*, 5 = *most deprived*).

**Results:**

There was no RSV transmission in 2020. In all other years, hospitalisation rates were highest for people of Pacific versus other ethnic groups and for people living in the most deprived quintile of households. RSV hospitalisation rates were higher in 2021 and 2022 than in 2018–19. The epidemic peak was higher in 2021, but not 2022, and the duration was shorter than in 2018–19. In 2021, the increase in RSV hospitalisation rates was significant across all age, sex, ethnic and socioeconomic groups. In 2022, the increase in hospitalisation rates was only significant in one age (1– < 3 years), one ethnic (Asian) and one socioeconomic group (quintile 2).

**Conclusions:**

COVID pandemic responses altered RSV‐related hospitalisation seasonal patterns. Atypical features of RSV hospitalisation epidemiology were the increase in rates in older children and young adults, which lessened in 2022. Despite these variations, RSV hospitalisations in NZ continue to disproportionately affect individuals of Pacific ethnicity and those living in more socioeconomically deprived households. Whilst future public health strategies focused on RSV disease mitigation need to consider the potential shifts in epidemiological patterns when the transmission is disrupted, these variances must be considered in the context of longer‐standing patterns of unequal disease distribution.

## 1 Background

Globally, respiratory syncytial virus (RSV) is a leading cause of acute respiratory tract infections (ARIs) amongst infants and young children [1]. It also has an important health and economic burden on adults, particularly older adults [2].

Stringent public health and social measures during the coronavirus disease 2019 (COVID‐19) pandemic greatly reduced the circulation of many respiratory viruses [3], including RSV [3, 4]. Following low RSV disease activity in 2020, unusual interseasonal RSV spikes or a higher‐than‐typical peak with a change in seasonality were reported in 2021 from both northern and southern hemisphere countries [5, 6, 7].

In the pre‐COVID‐19 pandemic, RSV infections followed a predictable annual seasonal pattern, consistently peaking in the winter in temperate northern regions and during rainy seasons in tropical and subtropical regions [8, 9]. RSV has no known animal reservoir; therefore, the viral transmission and seasonality depend on human environmental, demographic and behavioural factors [10, 11, 12].

Several factors are likely to have contributed to the changes in RSV epidemiology that occurred immediately following the COVID‐19 pandemic. These include changing population immunity due to the absence of RSV circulation, COVID‐19‐induced immune dysregulation, interactions between SARS‐CoV‐2 and RSV, changes in healthcare system organisation and changes in health‐seeking behaviour [13].

In NZ before 2020, RSV displayed annual seasonality. It was responsible for approximately 40% of ARI hospitalisations amongst children < 5 years old during the winter seasons 2012 through 2015 [14]. RSV disproportionally affects population groups defined by ethnicity and socioeconomic status (SES). In a previous NZ study, being of Māori (NZ's indigenous population) or Pacific ethnic groups or living in a neighbourhood with low SES were independently associated with increased RSV hospitalisation rates for children and those aged > 18 years [14].

NZ implemented a nationwide lockdown from 25 March to 27 April 2020 as part of its COVID‐19 elimination strategy. This included border closure to non‐New Zealanders and 14‐day quarantine for returning travellers; widespread testing, isolation of cases, contact tracing and quarantine of exposed persons; physical distancing recommendations; and individual infection prevention and control measures and communicating risk to the public and various stakeholders [15, 16]. In the postlockdown period of 2020, laboratory‐based surveillance showed a marked reduction in viral‐related respiratory illness, including eliminating community transmission of influenza and RSV [17]. In April 2021, the NZ border closure was eased to allow exclusive movement between Australia and NZ. In June 2021, NZ surveillance reported a surge in RSV cases [17].

Within this context, we report the epidemiology of RSV cases in NZ before and after the onset of the COVID‐19 pandemic. Data sourced from multiple surveillance platforms were combined for analysis with the pattern of RSV infection stratified by demographic factors (age, sex, ethnicity and SES). This analysis provides additional demographic data that complements existing reports of RSV epidemiology after a period of disruption in transmission.

## 2 Methods

### 2.1 Study Population

This analysis included people of all ages who presented to hospitals with a severe acute respiratory infection (SARI) and registered within the established NZ surveillance platforms during the study intervals 2018 and 2022 [18, 19].

Since 2012, active population‐based surveillance for SARI has been conducted in the four NZ hospitals (two adult and two paediatric), which provide all acute inpatient care for the population living in the central, eastern and southern areas of Auckland, NZ (Auckland and Counties‐Manukau District Health Boards (ADHB and CMDHB)) [18, 19]. The catchment area has a population of approximately 1,100,000, of whom 12.7% are Māori (NZ's indigenous people), 17.5% are of Pacific ethnicities (including ethnic groups from Samoa, Cook Islands, Tonga, Niue, Fiji, Tokelau, Tuvalu and Kiribati), 30.1% are Asian and 39.7% are NZ European or other ethnicities [20]. In NZ, the term Asian refers to those with East, South and Southeast Asia origins. This term in NZ excludes those from the Middle East, Russia and Central Asia; a line is made at either Pakistan or Afghanistan, with any countries to the north or west excluded. Statistics NZ use this definition of ‘Asian’, which is demonstrated in the NZ census [21]. Active surveillance consisted of a research nurse review of daily records of all overnight acute admissions to identify patients with ARI symptoms. The nurses enrolled those who met the WHO SARI case definition: an illness with a history of fever or measured fever ≥ 38°C and cough with onset in the preceding 10 days [22]. A respiratory specimen was collected (nasopharyngeal or nasal or throat swab) from each SARI case and tested for influenza and other respiratory viruses, including RSV, using real‐time reverse transcription polymerase chain reaction (PCR) techniques [19].

### 2.2 Statistical Methods

Two or more SARI hospitalisations within 14 days, including transfers to other facilities, were considered the same episode. Weekly counts of SARI hospitalisations and RSV virus detections are presented as a figure. A descriptive analysis of characteristics of total SARI and RSV‐associated SARI (RSV‐SARI) hospitalisations by demographic variables was presented as numbers and percentages in Table 1. Incidence rates (IRs) were calculated by dividing the number of RSV‐SARI hospitalisations (singular episodes) by the number of people residing in the study area. Confidence intervals for IRs and rate ratios (RRs) were based on the Poisson distribution. Rates were stratified by age, sex, prioritised ethnicity (Māori, Pacific, Asian or European/other) and SES, as they are considered key modifiers of SARI hospitalisation. The denominator was the residents of ADHB or CMDHB hospital catchment areas during the study period whose SES, as estimated by the 2018 NZ Deprivation Index, a small‐area composite measure [23], was used to separate the study sample into SES quintiles. The New Zealand Deprivation Index (NZ Dep 2018) reflects eight dimensions of material and social deprivation, which reflect a lack of income, employment, communication, support, qualifications, home ownership, living space and dry living conditions. The ordinal scale ranging from one to five (quintiles) was used, where one represented the areas with the least deprived index scores and five with the most deprived index scores. For SARI cases, the SES data were obtained from the NZ Ministry of Health (MoH).

**TABLE 1:** SARI cases and RSV‐associated hospitalizations by demographic characteristics and year, Auckland, NZ, during the two pre‐COVID years (2018, 2019) and the two post‐COVID lockdown years (2021, 2022).

	2 years, 2018 and 2019			The year 2020	Year 2021				Year 2022			
	Total SARI Events (*n*)	RSV (*n*)	% tested positive (row), 95% CI^c^		Total SARI Events (*n*)	Tested	RSV (*n*)	% tested positive (row), 95% CI	Total SARI Events (*n*)	Tested	RSV (*n*)	% tested positive (row), 95% CI
Overall	1429^a^	255	17.9 (16.0–19.9)		912	822	423	51.5 (48.0–54.9)	1519	1346	117	8.7 (7.3–10.3)
Age in years												
< 1	426	176	41.3 (36.7–46.1)		226	219	172	78.5 (72.6–83.5)	238	238	60	25.2 (20.1–31.1)
1 to < 3	136	36	26.5 (19.7–34.5)		183	170	119	70.0 (62.7–76.4)	232	231	42	18.2 (13.7–23.7)
3 to < 5	44	14	31.8 (19.8–46.8)		58	50	24	48.0 (34.6–61.7)	62	62	5	8.1 (3.4–18.0)
5–19	55	2	3.6 (0.9–13.4)		47	44	17	38.6 (25.5–53.6)	92	90	2	2.2 (0.6–8.5)
20–49	188	3	1.6 (0.5–4.9)		109	95	28	29.5 (21.2–39.4)	245	197	2	1.0 (0.3–4.0)
50–64	207	8	3.9 (2.0–7.6)		97	81	17	21.0 (13.5–31.2)	198	160	1	0.6 (0.1–4.3)
65–79	232	7	3.0 (1.4–6.2)		120	104	28	26.9 (19.3–36.2)	282	240	5	2.1 (0.9–4.9)
80+	141	9	6.4 (3.4–11.8)		72	59	18	30.5 (20.1–43.3)	169	127	0	0.0 (0.0–0.0)
Sex												
Female	714	120	16.9 (14.3–19.8)		444	399	210	52.6 (47.7–57.5)	749	662	65	9.8 (7.8–12.3)
Male	710	134	18.9 (16.2–21.9)		467	422	213	50.5 (45.7–55.2)	769	684	52	7.6 (5.8–9.8)
Ethnicity												
European/other	320	34	10.6 (7.7–14.5)		249	218	107	49.1 (42.5–55.7)	440	368	26	7.1 (4.9–10.2)
Māori	391	78	20.0 (16.3–24.3)		201	188	96	51.1 (43.9–58.2)	252	240	27	11.3 (7.8–15.9)
Pacific	581	118	20.3 (17.3–23.8)		319	295	162	54.9 (49.2–60.5)	602	543	42	7.7 (5.8–10.3)
Asian	137	25	18.2 (12.6–25.6)		143	121	58	47.9 (39.2–56.8)	225	195	22	11.3 (7.5–16.5)
SES^b^												
1 (highest quintile)	39	6	15.4 (7.1–30.3)		40	36	21	58.3 (41.9–73.1)	88	74	5	6.8 (2.8–15.2)
2	86	9	10.5 (5.5–18.9)		102	90	51	56.7 (46.3–66.5)	156	133	12	9.0 (5.2–15.2)
3	78	12	15.4 (8.9–25.2)		96	77	39	50.6 (39.6–61.6)	168	135	9	6.7 (3.5–12.3)
4	227	48	21.1 (16.3–26.9)		189	164	91	55.5 (47.8–62.9)	296	261	20	7.7 (5.0–11.6)
5 (lowest quintile)	985	178	18.1 (15.8–20.6)		483	453	221	48.8 (44.2–53.4)	803	736	69	9.4 (7.5–11.7)

*Note:* The total numbers in each category may not be the same due to missing values.

^a^ *n* = 1427 were tested.

^b^ Socioeconomic status (SES) is based on a small area‐level measure of household deprivation derived from the national census (NZ Dep 2018), an ordinal scale of 1–10 where 1 indicates the individual living in a household with the least socioeconomic deprivation. We used the ordinal scale ranging from one to five (quintile), where one represented the areas with the least deprived scores and five with the most deprived scores.

^c^ CI, confidence interval.

Poisson regressions with crude model and model adjusted for age, ethnic groups and SES were used to compare RSV‐related hospitalisation rates using RRs (Table 2). Composite variables for the ethnic group (Māori, Pacific peoples, Europeans and others, including Asians) and SES (highest quintiles [one to three] and lowest quintiles [four to five]) were created to facilitate the assessment of any modification of effects between ethnicity and SES. As testing for RSV occurred in 93.5% of SARI hospitalisations, modelling of weekly SARI counts and RSV virus detections is presented here without adjustment for missing data. Study data were captured using the REDCap 10.0.19 electronic data capture tool, with data exported for analysis in Stata 16.1 (StataCorp LLC).

**TABLE 2:** The crude and adjusted incidence rate ratios of severe acute respiratory infections associated with RSV by age, ethnic and socioeconomic group in Auckland, New Zealand, in 2021 and 2022 compared with 2018 and 2019.

	2 years, 2018 and 2019		Year 2020	Year 2021		Year 2022	
	Crude IRR^a^ (95% CI)	Adjusted^b^ IRR (95% CI^c^)		Crude IRR (95% CI)	Adjusted IRR (95% CI)	Crude IRR (95% CI)	Adjusted IRR (95% CI)
Age group in years							
< 1	2047.9 (654.1–6411.5)	1561.8 (498.5–4892.8)		225.3 (151.1–336.0)	194.7 (130.4–290.5)	1072.1 (262.0–4387.8)	954.5 (232.9–3911.0)
1 to < 3	207.3 (63.8–674.1)	155.9 (47.9–507.1)		76.0 (50.3–114.6)	68.2 (45.2–103.0)	384.5 (93.1–1588.20)	349.7 (84.6–1445.9)
3 to < 5	81.20 (23.3–282.7)	61.1 (17.5–212.8)		15.2 (8.8–26.3)	13.5 (7.8–23.4)	35.4 (6.5–193.3)	33.1 (6.0–180.6)
5–19	1.5 (0.2–8.8)	1.1 (0.2–6.4)		1.3 (0.7–2.4)	1.1 (0.6–2.1)	2.2 (0.3–15.4)	2.0 (0.3–13.9)
20–49	Ref	Ref		Ref	Ref	Ref	Ref
50–64	7.2 (1.9–27.1)	8.0 (2.1–30.2)		1.6 (0.9–2.9)	1.6 (0.9–3.0)	1.30 (0.10–13.90)	1.3 (0.1–14.8)
65–79	12.1 (3.1–46.9)	15.3 (3.9–59.2)		4.7 (2.8–7.9)	5.2 (3.1–8.9)	11.3 (2.2–58.4)	12.4 (2.4–64.2)
80+	54.0 (14.6–199.4)	77.4 (20.8–288.1)		10.3 (5.7–18.7)	11.7 (6.4–21.4)	0.0	0.0
Ethnic group							
European/other	Ref	Ref		Ref	Ref	Ref	Ref
Māori	6.9 (4.6–10.3)	2.2 (1.4–3.5)		2.7 (2.0–3.5)	1.4 (1.0–1.9)	3.0 (1.7–5.1)	1.0 (0.5–1.7)
Pacific	8.1 (5.5–11.9)	2.4 (1.6–3.7)		3.4 (2.7–4.4)	1.8 (1.3–2.4)	3.4 (2.0–5.5)	1.1 (0.6–1.9)
Asian	1.0 (0.6–1.7)	0.6 (0.4–1.1)		0.7 (0.5–1.0)	0.6 (0.4–0.8)	1.1 (0.6–1.9)	0.7 (0.4–1.2)
Socioeconomic status group							
1 (highest quintile)	Ref	Ref		Ref	Ref	Ref	Ref
2	1.3 (0.5–3.6)	1.2 (0.4–3.4)		2.1 (1.3–3.5)	2.1 (1.3–3.5)	2.1 (0.7–5.9)	2.1 (0.7–6.0)
3	1.7 (0.6–4.5)	1.6 (0.6–4.2)		1.6 (0.9–2.7)	1.6 (0.9–2.7)	1.5 (0.5–4.5)	1.6 (0.5–4.8)
4	7.1 (3.1–16.7)	4.8 (2.0–11.4)		3.9 (2.4–6.3)	3.2 (2.0–5.3)	3.6 (1.3–9.6)	3.1 (1.1–8.4)
5 (lowest quintile)	17.1 (7.6–38.5)	7.1 (3.1–16.4)		5.9 (3.8–9.3)	3.4 (2.1–5.4)	7.7 (3.1–19.0)	5.1 (2.0–13.1)

^a^ IRR: Crude incidence rate ratio.

^b^ Adjusted IRR: Rate ratio adjusted for age, ethnicity and socioeconomic status.

^c^ CI, confidence interval.

^d^ SES: Socioeconomic status (SES) based on a small area‐level measure of household deprivation derived from the NZ national census (NZ Dep 2018) where 1 indicates the individual is living in a household that is in the least socioeconomic‐deprived quintile and 5 indicates an individual living in a household that is in the most socioeconomic‐deprived quintile of all NZ households.

### 2.3 Ethical Approval

Ethical approval was obtained for SHIVERS (including SARI and ILI/ARI surveillance), SHIVERS‐II, III and IV cohort studies; and SHIVERS‐V surveillance from the NZ Northern A Health and Disability Ethics Committee (NTX/11/11/102). The laboratory‐based respiratory virus surveillance data forms part of standard public health surveillance in NZ. This surveillance is conducted in accordance with the NZ legislation, the Public Health Act, and thus, ethics committee approval was not needed for the collection or use of these data.

## 3 Results

### 3.1 Trends in RSV‐SARI Hospitalisations

**FIGURE 1:**
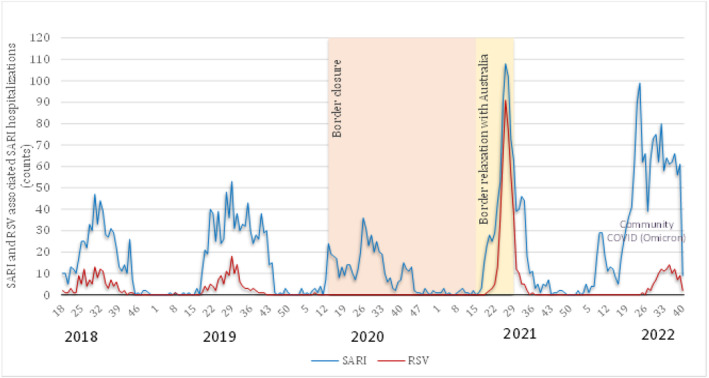
Weekly counts of severe acute respiratory infection (SARI) cases and respiratory syncytial virus (RSV)‐associated SARI cases from 2018 to 2022.

The established seasonal pattern for RSV‐SARI hospitalisations during the southern hemisphere winter in NZ was disrupted after the onset of the SARS‐CoV‐2 pandemic. During 2020 year‐round surveillance, no RSV cases were detected. In 2021, RSV cases showed a marked seasonality pattern, but with a more rapid rise and decline and higher peak incidence, peaking in week 26 (1^st^ week in July 2021) and sharply decreasing 5 weeks later in week 31. In contrast, the following year, 2022, had a similar pattern to 2018 and 2019, with a slower rise and fall of cases and a peak that was lower than the 2021 peak but higher than the peaks that occurred in 2018 and 2019 (Figure 1).

### 3.2 Characteristics of Total SARI and RSV‐SARI Hospitalisations

According to the results presented in Table 1, there were 255 and 423 RSV‐associated hospitalisations in 2018–2019 and 2021, respectively, and the detection rate in 2021 was about three times greater (51% vs. 17.9%) than in 2018–2019 (Table 1). There were increases in cases in all age, ethnic and socioeconomic groups. Notably, in 2021, only 40% of all RSV‐SARI cases were in children < 1 year old compared to 70% during 2018–2019. In 2022, although all‐cause SARI hospitalisations increased, RSV‐SARI cases decreased in number to approximate the annual number of cases seen in 2018 or 2019.

### 3.3 RSV Hospitalisation Patterns

Compared with 2018 and 2019, RSV‐associated hospitalisation rates varied in 2021 and 2022 (Table 3). The increase in hospitalisation rates in 2021 was significant in all ages, sex, ethnic and socioeconomic groups. In 2022, the pattern of RSV‐associated hospitalisations was different from 2021, and overall, changes in IR were not much different from the 2018/2019 figures.

**TABLE 3:** RSV‐associated hospitalizations rate by age group, sex, ethnic group and socioeconomic status in Auckland, New Zealand, in 2021 and 2022 versus 2018 and 2019.

	2 years, 2018 and 2019	2020	2021		2022	
	Crude IR^a^ (95% confidence interval [CI])		Crude IR (95% CI)	Changes in IR^b^ 2021 vs. 2018/19	Crude IR (95% CI)	Changes in IR 2022 vs. 2018/19
Overall	11.7 (10.3–13.1)		38.5 (34.8–42.1)	3.3 (2.8–3.8)	10.5 (8.6–12.5)	0.9 (0.7–1.1)
Age group in years						
< 1	631.6 (537.7–725.4)		1297.2 (1103.4‐1491.1)	2.1 (1.7–2.5)	448.4 (334.0–562.8)	0.7 (0.5–1.0)
1 to < 3	63.9 (42.8–85.1)		437.3 (358.7–515.8)	6.8 (4.7–10.0)	160.8 (112.2–209.4)	2.5 (1.6–3.9)
3 to < 5	25.1 (11.9–38.2)		87.7 (52.6–122.8)	3.5 (1.8–6.8)	14.8 (0.3–29.3)	0.6 (0.2–1.8)
5–19	0.5 (−0.2–1.1)		7.6 (4.0–11.3)	16.8 (3.9–72.9)	0.9 (−0.3–2.2)	2.0 (0.3–14.2)
20–49	0.3 (0.0–0.7)		5.8 (3.6–7.9)	18.7 (5.7–61.4)	0.4 (−0.2–1.0)	1.4 (0.3–8.1)
50–64	2.2 (0.7–3.8)		9.0 (4.7–13.3)	4.0 (1.7–9.4)	0.5 (−0.5–1.6)	0.2 (0.03–1.9)
65–79	3.7 (1.0–6.5)		27.1 (17.0–37.1)	7.2 (3.2–16.6)	4.7 (0.6–8.9)	1.3 (0.4–4.0)
80+	16.7 (5.8–27.5)		59.4 (32.0–86.8)	3.6 (1.6–7.9)	0.00	0.00
Sex						
Female	11.0 (9.0–13.0)		38.1 (32.9–43.2)	3.5 (2.8–4.3)	11.7 (8.8–14.5)	1.1 (0.8–1.4)
Male	12.4 (10.3–14.5)		38.8 (33.6–44.1)	3.1 (2.5–3.9)	9.4 (6.8–11.9)	0.8 (0.5–1.0)
Ethnic group						
European/other	3.9 (2.6–5.3)		24.7 (20.0–29.3)	6.3 (4.3–9.2)	6.1 (3.8–8.5)	1.6 (0.9–2.6)
Māori	27.2 (21.1–33.2)		65.6 (52.5–78.7)	2.4 (1.8–3.3)	18.3 (11.4–25.1)	0.7 (0.4–1.0)
Pacific	31.9 (26.10–37.7)		84.7 (71.6–97.7)	2.7 (2.1–3.4)	20.6 (14.2–27.0)	0.6 (0.5–0.9)
Asian	3.9 (2.4–5.4)		17.7 (13.1–22.2)	4.5 (2.8–7.2)	6.7 (3.9–9.5)	1.7 (1.0–3.1)
Socioeconomic status group						
1 (highest quintile)^c^	1.7 (0.3–3.1)		11.9 (6.8–16.9)	6.9 (2.8–17.1)	2.9 (0.4–5.4)	1.7 (0.5–5.5)
2	2.2 (0.8–3.7)		24.9 (18.1–31.7)	11.2 (5.5–22.7)	5.9 (2.6–9.3)	2.7 (1.1–6.3)
3	2.9 (1.3–4.6)		18.6 (12.8–24.4)	6.4 (3.3–12.2)	4.3 (1.5–7.20)	1.5 (0.6–3.5)
4	12.3 (8.80–15.8)		46.6 (37.0–56.2)	3.8 (2.7–5.4)	10.3 (5.8–14.8)	0.8 (0.5–1.4)
5 (lowest quintile)	29.4 (25.10–33.7)		70.6 (61.3–79.9)	2.4 (2.0–2.9)	22.0 (16.8–27.2)	0.7 (0.6–1.0)

^a^ IRs (incidence rates) were calculated by dividing the number of RSV‐associated SARI hospitalizations (singular episodes) by the number of people residing in the study area (residents of ADHB or CMDHB hospital catchment areas during the study period).

^b^ Changes in IRs: Incidence rates in 2021 and 2022 were compared to their figures in 2018/2019 as a reference year, and socioeconomic status based on a small area‐level measure of household deprivation derived from the NZ national census (NZ Dep 2018) where 1 indicates the individual is living in a household that is in the least socioeconomic‐deprived quintile and 5 indicates an individual living in a household that is in the most socioeconomic‐deprived quintile of all NZ households.

^c^ Please note that all numbers have been rounded to the nearest; changes might be different if rounded numbers are used directly.

In 2021, concerning age groups, the change in adjusted IR was smallest in children < 1 year old (IR = 2.1) and largest in children and adolescents aged 5–19 years (IR = 15.2) and adults aged 20–49 years (IR = 19.3). The increase in hospitalisation rates in 2021 was evident in all ethnic groups. Still, it was larger in those of European or other ethnic groups (IR = 6.3) and Asian group (IR = 4.5) than in Māori (IR = 2.4) or Pacific (IR = 2.7). The increase in hospitalisation rates in 2021 was significant in all socioeconomic quintiles but was smaller in the two most deprived quintiles (quintile 5 IR = 2.4, quintile 4 IR = 3.8) and largest in quintile 2 (IR = 11.2).

In 2022, concerning age groups, the change in IR was only significant for children aged 1– < 3 years (IR = 2.5). The increase in hospitalisation rates in 2022 was significant for the Asian ethnic group (IR = 1.7) and those of European or other ethnic groups (IR = 1.6). The increase in hospitalisation rates in 2022 remained statistically significant for quintile 2 (IR = 2.7).

### 3.4 Multivariable Modelling of RSV SARI

In all three time periods, in a multivariable model including age, ethnicity and SES groups, the highest risk of RSV infection was observed amongst children < 1 year old, with the risk decreasing up to age 20–49 years and then increasing again as the age group increased. In 2021, as in 2018/19, the adjusted IRR was increased for Māori and Pacific compared with European/others. However, in 2022, there was no difference in IRR by ethnic group. In all three time periods, the highest risk of RSV infection was observed amongst those living in households in quintile 5, with the risk also increasing for those living in households in quintile 4. In addition, in 2021, the adjusted IRR for those living in households in quintile 2 was increased (IRR = 2.1).

**FIGURE 2:**
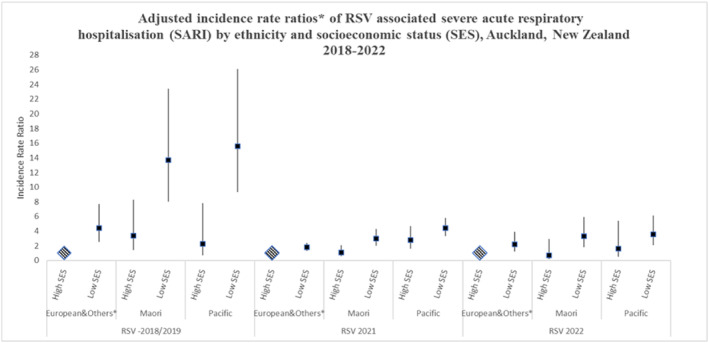
Adjusted incidence rate ratios of respiratory syncytial virus.

When considering SES within ethnic groups and after adjustment for age, the differences in RSV hospitalisation rates by SES and ethnic group were more evident, with the SES difference in incidence rate predominantly amongst individuals of Māori and Pacific ethnic groups (Figure 2).

## 4 Discussion

### 4.1 Statement of Principal Findings

In this active surveillance study of RSV‐SARI hospitalisations during the 2 years before and 2 years after the elimination of RSV circulation through border closures and social isolation strategies, differences in the epidemiology of RSV‐SARI hospitalisations were observed in both years following the reopening of borders but differed in these 2 years. Overall, RSV hospitalisation rates were higher in the years when RSV circulation returned than in those prior. Other important differences were a shorter epidemic duration and a higher peak for 1 year after RSV returned, but not the following year. When the disease returned, the increases seen in RSV hospitalisation rates were significant in all age, sex, ethnic and socioeconomic groups. In the 2nd year post‐return, these changed remained only significant for infants aged 1 to under 3 years, those of Asian ethnicity and one socioeconomic group (quintile 2).

There were also significant changes by ethnicity; consistently, hospitalisation rates were highest for people of Pacific versus other ethnic groups and for people living in the most deprived quintile of households. However, after a period of no transmission, the increase in hospitalisation rates is larger in those of European or other ethnic groups. The increase in hospitalisation rates was also significant in all socioeconomic quintiles but smaller in the two most deprived quintiles. The changes did not all persist through to the year following, with the increase in hospitalisation rates being significant only for the Asian ethnic group and people living in quintile two households.

### 4.2 Strengths and Limitations of the Study

This study is the first to provide insight into the temporal changes in RSV epidemiology occurring in the post‐COVID pandemic lockdown era in New Zealand. It was conducted in a country which implemented a lockdown of sufficient intensity to eradicate community RSV transmission. It allowed us to demonstrate robust data on RSV changes through active integrated population‐based surveillance for SARI in patients admitted overnight.

Our study has several limitations. The first was only based on hospitalised individuals and did not cover moderate to mild cases in the community. Second, the analysis does not consider the RSV subtype. Without RSV subtyping, we cannot detect which subgroup was predominant or more severe during the study period. Third, we were unable to establish whether COVID‐19 effects impacted the rates of hospital admission.

### 4.3 Strengths and Weaknesses in Relation to Other Studies, Discussing Particularly Any Differences in Results

Unlike many counties, this NZ study was enabled by active surveillance, which has been underway in NZ since 2012. The surveillance methods used were identical across the post‐ and pre‐COVID lockdowns, which provided robust denominator estimates.

### 4.4 Unanswered Questions and Future Research

Secondary to the introduction and easing of measures designed to limit SARS‐CoV‐2 transmission, the seasonal patterns for respiratory viruses were disrupted [24]. Disruption to the RSV seasonal epidemics has been reported in many high‐income countries, including Denmark [25], Spain [26], England [27], Australia [28], France [29], Germany [30], Saudi Arabia [31] and the USA [32]. Following this period of disruption, the return of the RSV epidemic pattern has been associated with out‐of‐season outbreaks [4, 5, 7, 26, 27, 28, 31, 32, 33], more rapid increases in hospitalisation rates during epidemics and higher overall incidence [4, 27]. Changes in epidemiological patterns by age group have occurred [7], but not necessarily with greater disease severity [34].

Like Australia, the NZ borders were essentially closed to the rest of the world from March 2020, with quarantine for country entrants applied during 2020. On 27 February 2021, an exclusive ‘travel bubble’ was opened between Australia and NZ. New Zealand's borders to the rest of the world remained essentially closed until July 2021.

Notably, Auckland Airport, the main international airport for NZ, is in the catchment area for this study cohort. Within weeks of easing the border restriction to Australia, NZ saw the reintroduction of RSV, which matched the standard seasonal timing. However, as described [25, 26, 27, 28, 29, 30, 31, 32], a more rapid epidemic rise with a higher peak was seen than in previous years.

With no recorded cases of RSV in all the NZ national surveillance for the years 2020 and early 2021 [24], the temporal association with cases being recorded just weeks after easing border restrictions only with Australia suggests that the timing of the onset was related to travel from Australia. Furthermore, genomic analysis of the strains confirmed their Australian origin.

RSV impacts most severely on young infants and older persons [1]. In the pre‐COVID era, there was a stable yearly epidemiological pattern with the highest burden, particularly in infants, followed by children < 5 years old and much older adults. Post the arrival of SARS‐CoV‐2 and the social responses to this leading to a 1‐year absence of recorded RSV cases in NZ, there was a dramatic shift in the age distribution. There was about a 19‐times increase in incidence for younger adults aged 20–49 years and approximately a 17‐times increase for children and adolescents aged 5–19 years old, with a comparatively lesser increase in incidence for younger children and infants. Changing epidemiological patterns by age group, particularly with the shift towards a greater burden for older children and younger adults, have been observed in other studies [25, 28, 30]. Suggested reasons for these changing patterns have focused on immunological naivety or diminished immunity from a population's lack of exposure in the previous season [25]. Whilst the concept of ‘immunity debt’ has been raised particularly about infants and young children [13, 27], this study suggests it is more around the shifting effects across a whole population. Potentially young infants may be more protected by some persisting maternal‐derived immunity compared to those aged 2–5 years who had limited contact with RSV; notably, some had been exposed pre‐2000. Older children and midlife adults may have previously had a lesser burden of hospitalisation due to repeated community exposure. Understanding the epidemiological impacts of the absence of circulation for one season is necessary to consider the role of regular exposure to RSV in older children and adults. Firstly, maintaining immunity through regular contact with the virus protects individuals against hospitalisation. Secondly, with these age groups repeatedly having regular exposure to asymptomatic or milder infection, this may reduce the burden of transmission across the whole population, reducing the impact of a rapid epidemic curve seen here in 2021.

The temporal epidemiological patterns observed in this study varied by ethnic and socioeconomic group. Previous research in NZ has documented that Māori and Pacific ethnic groups and people living in regions of the highest socioeconomic deprivation have significantly higher rates of hospitalisation for all acute respiratory illnesses, including those caused by RSV [34, 35]. Whilst Māori and Pacific populations and those living in households in more socioeconomically deprived areas continued to have a higher incidence rate in the post‐COVID era, relative differences were smaller than in the pre‐COVID era. We have been unable to identify any published data from other countries that have described the relative effects of a period of disease absence on the more vulnerable members of a population. The drivers for these changes are likely interrelated and multidimensional [36, 37]. For example, did lack of exposure to RSV in 2020 then later manifest as more severe disease in subgroups of the population who were traditionally less at risk of severe disease and had repeated exposure to the virus to maintain immunity? It is known that RSV spreads more in household crowding and larger households [13, 30]. Māori and Pacific families are more likely to have larger and more crowded households [38]. Therefore, with more regular exposure to the virus, a 1‐year absence of exposure may have a lesser effect in these situations where immunity has already been more frequently boosted.

What has been unclear internationally is the expected pattern of disease post the disruption from the COVID pandemic. Our study highlights that NZ's second seasonal epidemic curve returned to a pattern more like that seen in the pre‐COVID era, with significant differences by age, ethnicity and social deprivation group persisting.

### 4.5 Meaning of the Study: Possible Mechanisms and Implications for Clinicians or Policymakers

Consistent with studies from other countries, data from NZ has shown a COVID lockdown‐related disruption to the seasonal RSV epidemic pattern, followed by a rapid resurgence with a higher peak and a change in epidemiological patterns. This study demonstrates that, following a disruption to transmission, the disease reappeared across the community following a lift in border restrictions. During the resultant epidemic, hospitalisation rates for the established high‐risk groups were less affected than other groups, which in the past had consistently lower hospitalisation rates (older children, young adults and European and other ethnic groups). This finding highlights the importance of considering further the role of regular population exposure to RSV, particularly for those in traditionally lower‐risk groups, to protect individuals and to reduce community exposure for the higher‐risk groups. With the imminent arrival of RSV vaccines for high‐risk groups, a more nuanced public health strategic approach is needed that considers multiple factors in combination. Firstly, the use of vaccines and passive immunisation targeted at high‐risk groups. Alongside this, the role of public health social measures can impact community transmission, particularly at times of high burden to the community and health services. Thirdly, recognising the importance of some amount of regular community exposure within population groups traditionally at lower risk of severe disease maintains a certain level of community immunity, which can reduce their individual risk and spread to those at higher risk. A more dynamic integrated strategy could reduce the impact on the most vulnerable, maintain population immunity and prevent overloading health services by minimising sudden peak surges in hospitalisation.

## 5 Conclusion

In the NZ setting, RSV had a stable epidemiological pattern of illnesses requiring hospitalisation, with the largest burden in young infants followed by the elderly, and higher incidence rates in indigenous ethnic groups and in those living in households in more deprived areas. Following the disruption to transmission that occurred with the COVID‐19 pandemic responses, these patterns remained. Still, there was also a significantly increased incidence in older children and younger adults in particular. The epidemiological patterns returned mostly to the prepandemic pattern the following year but with still some atypical features. Despite these variations, RSV hospitalisations in NZ continued to disproportionately affect individuals of Māori and Pacific ethnicity and those living in more socioeconomically deprived households.

This study highlights the importance of being aware of the multiple dynamics of community exposure and transmission and the differing effects across the whole community when RSV transmission is temporarily interrupted. Future public health strategies focused on RSV disease mitigation must consider the potential shifts in epidemiological patterns when transmission is disrupted.

## Author Contributions

**Turner Nikki:** conceptualization, investigation, methodology, validation, visualization, writing–original draft, writing–review and editing, supervision. **Aminisani Nayyereh:** conceptualization, data curation, investigation, methodology, project administration, writing–original draft, writing–review and editing. **Huang Sue:** conceptualization, investigation, methodology, supervision, validation, visualization, writing–original draft. **O‐Donnell Jane:** writing–review and editing. **Trenholme Adrian:** visualization, writing–review and editing. **Broderick David:** validation, visualization. **Paynter Janine:** data curation, formal analysis, investigation, methodology, supervision, validation, visualization. **Castelino Lorraine:** project administration, resources. **Grant Cameron:** validation, visualization; writing–review and editing. **McIntyre Peter:** writing–review and editing, conceptualization, investigation, supervision, validation, visualization.

## Conflicts of Interest

The authors declare no conflicts of interest.

### Peer Review

The peer review history for this article is available at https://www.webofscience.com/api/gateway/wos/peer‐review/10.1111/irv.13346.

## Data Availability

The data supporting this study's findings are available on request from the first author. However, due to privacy or ethical restrictions, they are not publicly available.
